# Data-Derived Modeling Characterizes Plasticity of MAPK Signaling in Melanoma

**DOI:** 10.1371/journal.pcbi.1003795

**Published:** 2014-09-04

**Authors:** Marti Bernardo-Faura, Stefan Massen, Christine S. Falk, Nathan R. Brady, Roland Eils

**Affiliations:** 1 Division of Theoretical Bioinformatics, German Cancer Research Center (DKFZ), Heidelberg, Germany; 2 Institute of Pharmacy and Molecular Biotechnology (IPMB), Bioquant, Heidelberg University, Heidelberg, Germany; 3 Lysosomal Systems Biology, German Cancer Research Center (DKFZ), Bioquant, Heidelberg, Germany; 4 Institute of Transplant Immunology, IFB-Tx, Hannover Medical School, Hannover, Germany; 5 German Center for Infectious Diseases (DZIF) TTU-IICH, Hannover, Germany; 6 Systems Biology of Cell Death Mechanisms, German Cancer Research Center (DKFZ), Bioquant, Heidelberg, Germany; 7 Department of Surgery, Heidelberg University Hospital, Heidelberg, Germany; National University of Singapore, Singapore

## Abstract

The majority of melanomas have been shown to harbor somatic mutations in the RAS-RAF-MEK-MAPK and PI3K-AKT pathways, which play a major role in regulation of proliferation and survival. The prevalence of these mutations makes these kinase signal transduction pathways an attractive target for cancer therapy. However, tumors have generally shown adaptive resistance to treatment. This adaptation is achieved in melanoma through its ability to undergo neovascularization, migration and rearrangement of signaling pathways. To understand the dynamic, nonlinear behavior of signaling pathways in cancer, several computational modeling approaches have been suggested. Most of those models require that the pathway topology remains constant over the entire observation period. However, changes in topology might underlie adaptive behavior to drug treatment. To study signaling rearrangements, here we present a new approach based on Fuzzy Logic (FL) that predicts changes in network architecture over time. This adaptive modeling approach was used to investigate pathway dynamics in a newly acquired experimental dataset describing total and phosphorylated protein signaling over four days in A375 melanoma cell line exposed to different kinase inhibitors. First, a generalized strategy was established to implement a parameter-reduced FL model encoding non-linear activity of a signaling network in response to perturbation. Next, a literature-based topology was generated and parameters of the FL model were derived from the full experimental dataset. Subsequently, the temporal evolution of model performance was evaluated by leaving time-defined data points out of training. Emerging discrepancies between model predictions and experimental data at specific time points allowed the characterization of potential network rearrangement. We demonstrate that this adaptive FL modeling approach helps to enhance our mechanistic understanding of the molecular plasticity of melanoma.

## Author Summary

Signal transduction pathways can be described as static routes, transmitting extrinsic signals to the nucleus to induce a transcriptional response. In contrast to this reductionist view, the emerging paradigm is that signaling networks undergo dynamic crosstalk, both in disease and physiological conditions. To understand complex pathway behavior, it is necessary to develop methods to identify pathway interactions that are active as a consequence of stimuli and, importantly, to describe their evolution in time. To that end, we developed a method relying on prior knowledge networks in order to predict signaling crosstalk evolution, in response to perturbation and over time. The challenge we addressed was to establish a method dependent on information related to the topology of reported interactions, and not their mechanistic characteristics, and at the same time complex enough to reproduce the behavior of the signaling intermediates. The work presented here demonstrates that such an approach can be used to predict mechanisms that melanoma uses to rearrange its signaling and maintain its abnormal proliferation upon treatment.

## Introduction

Curated signaling networks are derived from reported interactions between proteins, including posttranslational modifications like phosphorylation. However, these interactions may be cell line dependent, occur at specific time points, or depend on context [1]. Moreover, the pathway of interest may be regulated by additional, unreported interactions. Such complexity is relevant in tumors, where signaling pathway rearrangements underlie resistance to the treatment, both via genetic mutations or epigenetic changes [2]. This treatment resistance is achieved in melanoma through its molecular plasticity, which includes neovascularization, migration [3], pathway rearrangement [4], and presence of subpopulations of cancer cells that may contain stem cell-like properties [5]. Specifically, resistance to treatment by small molecules has been reported to be developed through switching among the serine threonine kinase BRAF isoforms to activate the MAPK pathway [4], [6], a signaling network which plays a major role in proliferation and is a very attractive target for therapy due to the fact that it harbors somatic mutations in the majority of melanomas [7], [8]. In addition, alternative splicing can be used by tumors to establish crosstalk between apoptotic and survival pathways, thereby rearranging signaling in order to develop protection against apoptosis. In work by Kurada et al., the authors show that MADD, a splice variant of IG20, is overexpressed in cancer cells and tissues and can specifically activate MAPKs through Grb2 and Sos1/2 recruitment to grant protection against apoptosis upon tumor necrosis factor-α (TNFα) treatment [9].

**Figure 1:**
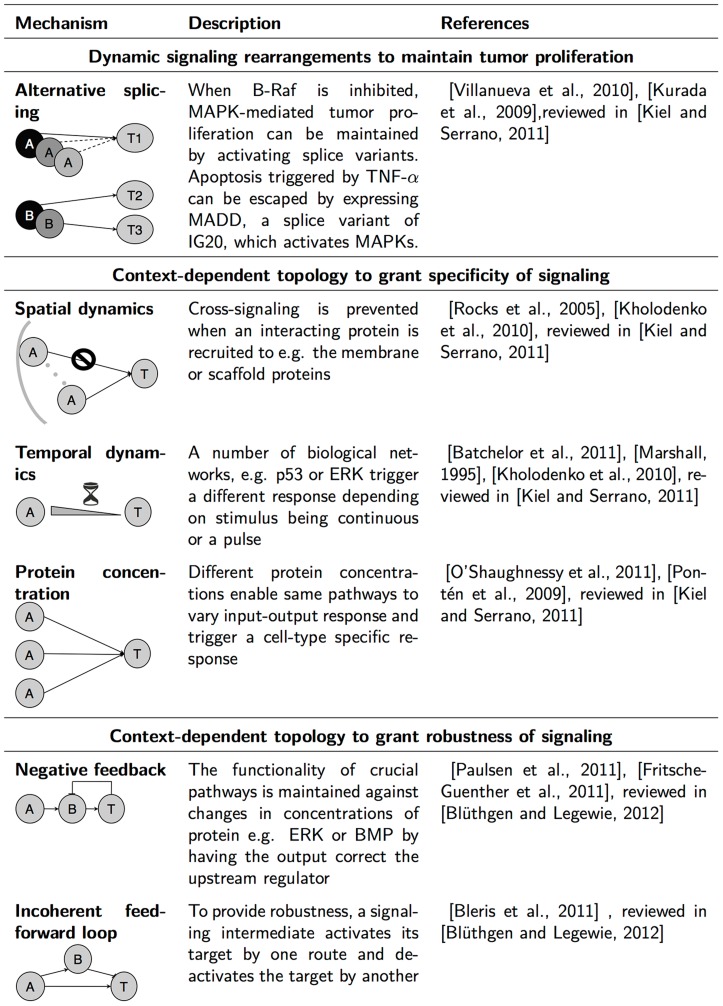
Overview of known mechanisms of MAPK signaling plasticity. Many reported interactions may not occur in the context of own experiments. Here, we review two types of mechanisms that yield differences between reported and experimental interactions. First, interactions can dynamically arise during observation to adapt to perturbation - here termed dynamic signaling rearrangements. Second, unreported interactions can be present from the unperturbed, onset of observation - here termed context-dependent topology. The upper row shows two known dynamic MAPK signaling rearrangements, which are achieved by preferentially expressing alternate transcripts to maintain tumor proliferation upon of treatment. In the middle section, three mechanisms are shown to change network topology in order to grant cells the ability to trigger a specific response to a ligand. Such specificity is achieved by tightly regulating known crosstalk interactions, thereby, preventing cross-signaling between pathways. Below, 3 rows describe different robustness mechanisms. When such mechanisms are present, an increase in the total protein concentration of a certain signaling regulator due to expression noise can be compensated to maintain the functionality of the pathway. The spectrum of mechanisms leading to both dynamic rearrangements and context-dependent topologies illustrates the need for methods to corroborate the activity of reported interactions in a timely and cell type specific manner.

On one hand, to identify those differences between reported and experimental signaling activated by a cancer cell to acquire resistance, it is necessary to study dynamic changes in signaling network topologies arising after perturbation. On the other hand, static differences are equally possible, with the reported and experimental topologies differing from the initial, unperturbed state of observation. This context-dependent network topology enables the cell to achieve important properties such as specificity of signaling and robustness of signaling. Indeed, the activation initiated by a ligand is not stably propagated through the full range of reported interactions in the corresponding cascade, since so many points of crosstalk exist that several unspecific responses could be activated [1]. Instead, a range of mechanisms enable the cell to enhance certain pathways or prevent some reported interactions from happening in order to trigger a specific response depending on the context or cell type [10], [11]. In addition, the threshold at which cells respond to stimulation present in a given context depends on the signaling pathway [12], and there exist several changes in the signaling network that can grant this robustness. In Figure 1, we describe dynamic and static changes in network topology according to the property that the cell can achieve by undergoing such changes. The existence of said mechanisms requires studying, rather than assuming, which amongst the reported interactions are active in the cell line used for experimental observation. In this work, we propose a method based on computational modeling to aid identification of both the dynamic and static topological changes in signaling that grant tumors its ability to maintain proliferation and develop resistance.

Several systems biology methods have been established to study network topology. In work by Ma et al., the authors provide a framework to define the range of network topologies that can achieve biochemical adaptation, where each network is represented by a system of Ordinary Differential Equations (ODEs) [13]. However, while this study provided insight into the motifs that can acquire adaptation, usage of ODEs requires vast knowledge on the specific reactions governing the interactions of the species [14]. Logic-based models offer one solution. In such formalisms, logic rules relate two species by using only the measurements of these two species and the information concerning their relation. Boolean logic has been used for functional analysis of extensive signal transduction networks [15], with the limitation that it assumes an on-off behavior of the modeled species. Fuzzy logic (FL) can predict intermediate states, utilize increased prior biological information, and thus improve content and accuracy of predictions.

A number of approaches based on FL have been used to encode signaling networks, and can be grouped in two major frameworks. In the first framework, models are constructed manually based on prior knowledge of topology and experimental measurements. Aldridge et al. established an approach to encode responses of colon cancer cells treated with combinations of pro-death and pro-survival cytokines, incorporating the role of time to model slow processes [16]. Within the same framework, FL was combined with other algorithms to represent the hedgehog-mediated regulation of the cell cycle [17]. This framework, however, has the limitation that one must manually calibrate FL models, which is a tedious task. In addition, manual parameterization requires extensive prior knowledge regarding the biochemical reactions included in the model, which is in several biological processes condition-dependent or unavailable. To overcome these limitations, the second framework consists of studies, in which the parameters of FL system are learned from data by means of training algorithms [18]. Using such an approach, we established a neuro-fuzzy-based method to reverse-engineer a potential hierarchy of interactions between mitochondrial morphological states and apoptotic events [19]. A limitation of these data-trained systems is that they require a high number of parameters, which grant the system its flexibility, but also increase the risk of over-fitting. As a solution, Morris et al. developed a constrained FL system that focused on specific states and applied it to elucidate interactions that were a priori possible but not present in the experimental data [20].

Here, we describe a data-derived modeling technique consisting of (i) a mathematical formalism, i.e. a generalization of a FL Inference System (which we termed gFIS) as the outcome of a strategy to reduce the number of free parameters, and (ii) a training and simulation pipeline. This approach can be used to characterize dynamic signaling rearrangements arising during observation to grant adaptation to treatment, as well as static context-dependent topologies, which are different from those reported in the unperturbed state. As a proof of concept, we used a dataset derived from a melanoma cell line exposed to different pharmacological kinase inhibitors, consisting of phosphorylated and total protein levels of 10 signaling intermediates involved in the MAPK pathway measured over 4 days. Next, we assembled a literature-based network of kinase signal transduction pathways containing the 10 readouts of our experiments. For each signaling intermediate in the network we created a gFIS model, which we calibrated using a subset of the data determined by the topology of the network. For instance, the model for CREB as regulated by P38 and ERK was trained using the normalized phospholevels measured for these three intermediates. Thereby, the prolonged non-linear behavior of the signaling network upon treatment was encoded. Subsequently, specific time points were removed from the training process and the performance of the trained network was evaluated in every iteration of this process. Finally, the model identified emergent literature-based versus model fit topological discrepancies in a time-dependent manner.

Overall, we demonstrate that novel insights in terms of signaling can be derived from time-defined FL training and simulation, allowing characterization of the time point of network rearrangement and can, therefore, be used to investigate the mechanisms that grant melanoma its molecular plasticity.

## Results

### Phosphorylation of MAPK signaling components over time

**Figure 2:**
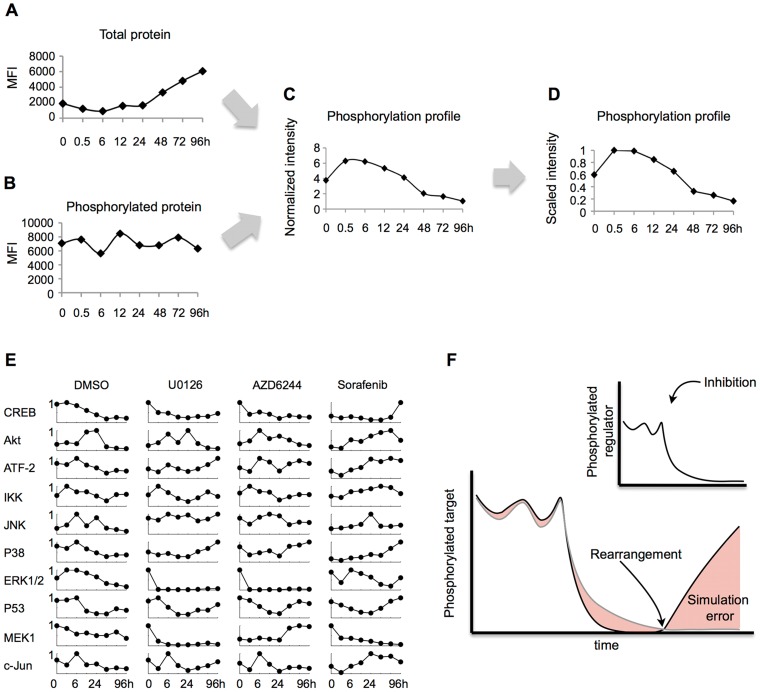
Strategy to study phosphorylation profiles in order to identify signaling rearrangements. Using bead-based multiplex analysis, the total and phosphorylated protein levels of 10 MAPK-related signaling intermediates and transcription factors were measured simultaneously at 0, 0.5, 6, 12, 24 36, 48, 72 and 96 hours in A375, a melanoma cell line with a constitutive MAPK activation driven by the BRAF V600E mutation. As an example, (**A**) total ERK1/2 and (**B**) phosphorylated ERK1/2 are depicted (MFI: Mean Fluorescence Intensity). (**C**) Phosphorylated protein levels were normalized to total, to account for total protein loss. (**D**) To enable model fitting and comparison, measurements were scaled to maximum protein value for each condition. (**E**) Full normalized dataset. Rows represent intracellular readouts assayed. The first column was measured in control conditions (DMSO). Columns 2 and 3 were acquired upon MEK-specific pharmacological inhibitors U0126 and AZD6244. The last column was acquired after treatment with multi-kinase inhibitor Sorafenib. (**F**) Cartoon illustrating the modeling approach presented here to identify rearrangements. When treated with a specific inhibitor, the phosphorylation of a regulatory kinase (black curve, upper panel) should be inhibited. Consistently, its target (black curve, lower panel) should be down-modulated. If signaling is rearranged, the target should stop responding to regulatory behavior, and a mathematical model (grey curve) assuming the original situation should exhibit an error increase (red area under the curve). The model expresses the target as a function of the regulator or regulators, hence inhibition, e.g. a phosphatase-substrate interaction, could equally be captured and revealed to be lost by error increase.

A375, a melanoma cell line featuring constitutive activation of the MAPK pathway due to the activating BRAF mutation V600E [21], was treated with three pharmacological kinase inhibitors. The transduction of the signal through the pathway was measured as level of phosphorylated protein for 10 signaling intermediates and transcription factors involved in the MAPK pathway at 8 time points spanning over 4 days. Therefore, we used the bead-based ELISA assays of xMAP technology (Luminex, Austin, TX) to measure the abundance of phosphorylated signaling intermediates and transcription factors including mitogen-activated protein kinase 1 (MEK1), extracellular signal-regulated kinase 1/2 (ERK1/2), cAMP response element-binding protein (CREB), protein kinase B/Akt (Akt), c-Jun n-terminal kinase (JNK), the JNK substrate c-Jun, IKK, P38 kinase, the cell cycle regulator P53 and the transcription factor ATF-2. Over such time ranges, expression and degradation events play an important role in the dynamics of the system [22]. Hence, abundance of the total proteins was also measured. Figure 2A shows measurements of total levels of ERK1/2 in control conditions and the level of the phosphorylated ERK1/2 proteins measured in the same sample is shown in Figure 2B.

Subsequently, phosphorylated protein levels were normalized to total in order to remove apparent loss of activation due to loss of total protein (Figure 2C). In the last step of our data processing pipeline, normalized values were scaled to the maximum value across all measurements in the same condition to avoid that higher intensity values could dominate the modeling process (Figure 2D). The phosphorylation state over 4 days of the full normalized dataset is shown in Figure 2E. Confirming drug potency, we observe that upon MEK-specific inhibitors U0126 and AZD6244 phosphorylation of ERK1/2 -the target of MEK1- is blocked, while upon multi-specific kinase inhibitor Sorafenib MEK1 phosphorylation decreases. As expected, a large number of signaling intermediates exhibit a constitutively high phosphorylation profile even though no stimulus was used, due to the BRAF V600E mutation. The fluctuations exhibited by a number of readouts can be due to the mechanisms described in Figure 1. For instance, it has been shown *in silico* that the combination of a negative feedback loop and ultrasensitivity can result in sustained biochemical oscillations [23]. Confirmation that the observed fluctuations are biochemical oscillations over such a broad time range would require higher density of measurements.

### Identifying emerging disagreement between prior knowledge based signaling networks and experimental data

We next developed a method to identify dynamic changes and differences between literature-based topologies and the regulatory network of interactions in the specific cell line of interest. The method is based on the assumption that if a signaling rearrangement occurs at a certain time point, the agreement between the involved intermediate and its substrates should greatly decrease after the given time point. Such disagreement should be revealed by an increase of the RMSE calculated for the model downstream of the rearrangement (Figure 2F). Therefore, the model should be able to capture directed regulation, i.e. not only activation but also inhibition. We subsequently describe the data-derived modeling strategy established to implement this concept. For clarification, Box 1 summarizes the terminology used hereafter. To illustrate the modeling process to encode a single intermediate and its behavior as a component of a signaling network, we considered the interaction between c-Jun and JNK. JNK is a member of the MAPK pathway that has been shown to be tyrosine and threonine phosphorylated as part of the stress and inflammatory response. Phosphorylated JNK can translocate to the nucleus to activate a number of transcription factors including c-Jun [24]. To map input data onto output data, IF-THEN logic rules lay at the core of logic models such as “*if phosphorylation of JNK is high then phosphorylation of c-Jun is high*”. Here, sets such as *high*, *medium*, or *low* are fuzzy sets. Conversely to Boolean logic, fuzzy sets have unsharp boundaries, i.e. measurements can belong to several fuzzy sets to a certain degree, requiring a transformation known as fuzzification [25] performed by the so-called membership functions (MF).

**Box 1. Summary of modeling terminology**

**Fuzzy Inference System (FIS)**: A model that uses fuzzy logic (FL) to infer an output based on an input. There are two main types of FIS: a linguistic model features logic rules with fuzzy sets such as *high* both in the premise and the consequent, e.g. “If JNK is high, then c-Jun is highly phosphorylated”. A Takagi-Sugeno model is a simplification of a Mamdani system, which uses fuzzy sets in its rule premise, but in its rule consequent is constrained to combinations of the inputs.

**Membership function (MF)**: A function to transform experimental data into fuzzy sets, thereby enabling use of FL rules.

**gFIS**: a *generalized* type of Takagi-Sugeno FIS established in this work, where a number of parameters and model qualities have been fixed, keeping as free parameters those enabling fitting to a generic dataset.

**mtFIS**: a multi-treatment FIS defined in this study. An mtFIS is the result of training a gFIS with *n* free parameters to multiple-treatment data, yielding a model with as many estimated parameters as *n* times the number of conditions. Given a number of regulators and one target, a trained mtFIS captures their relation and expresses the behavior of the target as a function of the regulators. Once a full signaling network has been trained, each node in the network is represented by one mtFIS.

**Figure 3:**
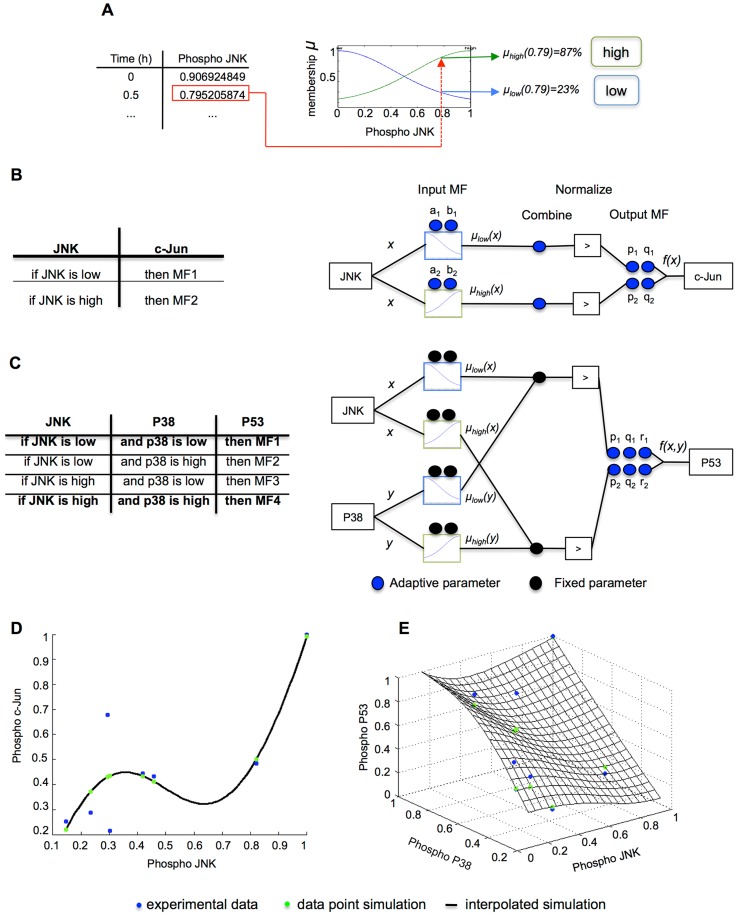
Parameter reduction strategy and data-derived model implementation. (**A**) JNK experimental data was transformed into fuzzy sets via 2 Gaussian input membership functions (MF), thereby enabling use of fuzzy logic rules. (**B**) The table shows logic rules mapping the phosphorylation of JNK to the phosphorylation of c-Jun, as an example. The diagram shows the parameters needed to implement those rules. 8 free parameters are required for the MFs (blue circles), which could increase for other types of function. In addition, the MFs to be combined in each rule need to be determined, here represented as 2 additional free parameters to a total of 10 in this simple FL system with two rules. (**C**) Implementation of a more complex FL model of P53 regulation by both P38 and JNK to illustrate the parameter reduction strategy. The number of consequent parameters depends on the number of rules, which in turn depend on the number of inputs, hence the setup described in (B) yields here 4 rules (table) with 20 parameters. To reduce the number of free parameters, we fixed the number of input-output combinations (left, in bold), reducing the system to 2 rules and 14 parameters (diagram). By fixing the premise parameters, the number of parameters to estimate was reduced to 6, below the number of experimental time points. (**D**) Simulation of the model shown in (B). The free parameters were reduced and estimated, and they proved to be sufficient to capture the data trend (blue dots) upon simulation (green dots, RMSE = 0.181), here illustrated in DMSO. The black curve shows the simulation of the model at 100 interpolated data points distributed uniformly. (**E**) Simulation of the model shown in (C), reduced and trained to the phosphorylation profiles of P38, JNK and P53. The simulation of P53 phosphorylation is depicted here as a mesh (RMSE = 0.223). Training data points are depicted in blue and simulation at the same data point in green. The black surface shows the simulation of the model at 100 interpolated data points distributed uniformly.

In previous work, we used Gaussian functions to fuzzify measurements of mitochondrial morphology and apoptotic signaling in order to explore their non-linear relationships in human breast carcinoma [19]. Analogously, input MFs were defined here as shown in equation 1 and used to establish the degree of membership of the JNK measurements –here represented as 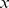
- to the sets *low* and *high* (Figure 3A).
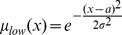
(1)Output MFs were defined as a first order polynomial, as established in first order Takagi-Sugeno systems (see methods for further detail). In this framework, logic rules establish which combination of input and output MFs are connected. Hence, a two-rules FL model 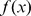 illustrated in Figure 3B can be expressed using the normalized outputs from the rule premise and a first-order polynomial for each rule as shown in equation 2.
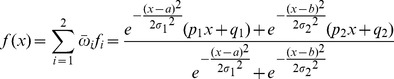
(2)Premise and consequent MF parameters can be learned from data [26], and these parameters can take negative values, enabling to account for opposite behavior such as an input being an inhibitor of the output modeled target.

In the shown model, it would be necessary to fit a, 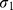, p_1_ and q_1_ for the first rule and b, 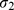, p_2_ and q_2_ for the second rule, to a total amount of 8 parameters solely for the definition of the MFs. Additionally, a list of model parameters can be learned from the data, e.g., variables related in each rule [27], number of rules [28] and type of MFs, increasing the number of parameters by orders of magnitude [18]. Importantly, an overly high degree of model complexity cannot be adequately met by experimental data typically leading to over-fitting when learning model parameters from data. Hence, we next reduced the number of parameters.

As the first step of our parameter reduction strategy, we fixed the number of fuzzy sets –and thereby the number of input MFs – to two, i.e. measurements were separated into being *low* and *high* to a certain degree. Next, the number of logic rules was fixed. As the number of rules depends on the number of inputs, to illustrate the reduction strategy we use an example FL system with two inputs, implemented to model P53. P53 has been shown to be activated by P38 kinase [29] and via JNK signaling [30]. To fix the number of rules we used a grid partition where all combinations of inputs MFs were allowed. Given that two fuzzy sets are defined for each input, the number of combinations was 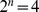, where n is the number of inputs (Figure 3C, table). Finally, the number of output MF parameters was fixed. Importantly, in Takagi-Sugeno systems, parameters of output MFs cannot be shared. Instead, in classical training different parameters are learned for each output MF [31]. Therefore, the above-mentioned system of rules would yield 3 consequent parameters for the linear combination of 2 inputs for each rule, i.e. 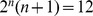 output parameters. Together with the 8 parameters for the input MFs, such a rule setup yields a total of 20 parameters. Therefore, a constraint was added to the reduction strategy, where only combinations of analogous MFs in all inputs where allowed. Thereby, the number of rules was fixed to the same number of fuzzy sets, yielding a system of 2 rules, 8 parameters of the input MFs and 6 parameters for the different output MFs summing to a total of 14 parameters (Figure 3C). In the last step of the parameter reduction strategy, we aimed at fixing the parameters themselves, as opposed to the above-mentioned steps to fix the number of parameters. Because the consequent parameters of each rule cannot be shared, we fixed the premise parameters (see materials and methods for the specific values and their rationale). Hence, the final number of parameters fitted is 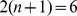 where n is the number of inputs. The system following the example shown in Figure 3C is expressed as 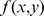 in equation 3

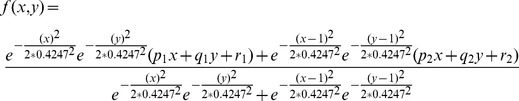
(3)Subsequently, we sought to assess whether the free parameters sufficed to capture the patterns in the data. Upon training, the JNK-c-Jun system (equation 2, illustrated in Figure 3B) showed a considerable accuracy, root-mean-square error (RMSE) = 0.181 (Figure 3D). For the JNK-P38-P53 system (equation 3, illustrated in Figure 3C), the data trend was captured as well (0.223, Figure 3E). Due to the high flexibility of the fuzzy inference system, several solutions could be found during the fitting process (see materials and methods for a detailed description of the objective function defined to select fits with best interpolation power).

Hence, the method established enabled to satisfactorily fit exclusively 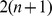 consequent parameters, where n is the number of inputs, thereby avoiding estimating (i) the number of rules, (ii) 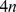 premise parameters and (iii) up to 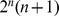 consequent parameters.

### Model complexity reduction retains model flexibility

**Figure 4:**
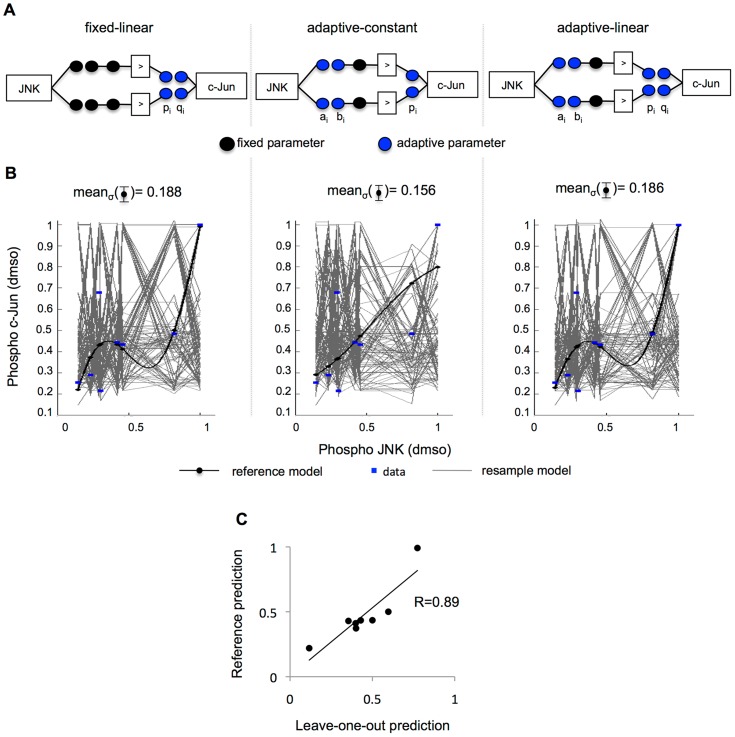
Data-derived sensitivity analysis confirms accuracy and flexibility of approach. Three formalisms were compared. (**A**) The schematic left represents the approach presented here, with fixed premise parameters a_i_, b_i_ and number of rules (black circles) and free linear consequent parameters p_i_ and q_i_ (blue circles) for each rule *i* (see equation 2). A zero-order Takagi-Sugeno fuzzy logic system is represented in the center schematic, which features the same input MFs –with free parameters here- and simpler consequent MFs, i.e a constant, with a single free parameter. The right-hand schematic shows the same setup as the left one, with the difference that no parameter was fixed. (**B**) 100 bootstrapped datasets, i.e. resampled with repetition, were used to train 100 models implemented with each setup (grey curves). The reference model was trained to the full original dataset (black curves for the model, blue dots for the experimental data). The standard deviation σ for the model simulation at each data point was calculated across the 100 bootstrapped models. As a signature of accuracy and flexibility, the mean of the deviation mean_σ_ for all data points was next calculated. While both the adaptive-linear and fixed-linear show an ability to adapt to the different datasets (mean_σ_(fixed-linear) = 0.188 and mean_σ_(adaptive-linear) = 0.186), the adaptive-constant setup exhibited a less flexible performance (center, mean_σ_(adaptive-constant) = 0.156) confirming that the consequent parameters outweigh the premise parameters in impact. (**C**) Leave-one-out cross-validation was performed to assess over-fitting by splitting the eight JNK-c-Jun pairs into two sets, leaving a single pair of observations as test set and the remaining time points as training set. A gFIS was fit to the training set and a prediction was made for the excluded observation using its JNK value for all eight data pairs, yielding one prediction for each differently trained model (x axis). Next, a gFIS was trained to the full dataset, creating a reference model that was used to make a prediction for each observed JNK value (y axis). Confirming model predictivity, the predictions of the reference model correlated largely with the test predictions (R = 0.89).

To successfully represent the behavior of a signaling intermediate solely as a function of the activity upstream and reveal time-defined disagreements (as illustrated in Figure 2E), the modeling approach established above had to be flexible, retaining its capability to adapt to changes in experimental data in spite of the reduction of free parameters. To validate this, we sought to assess the sensitivity of the data-derived model. One approach to sensitivity analysis is to directly modify the model parameters and subsequently measure model outcome. Instead, due to the data-derived nature of our approach, here the training data was resampled, which in turn led to modification of the fitted parameters. To study the effect of our reduction strategy on the flexibility of the system, the analysis was performed on the fully reduced setup (represented in Figure 4A, left) and two partially reduced systems where the number of rules and MFs was fixed but not the premise parameters: (i) the equivalent 0-order Takagi-Sugeno system (Figure 4A, center), featuring free parameters for the premise and free constant parameters instead of linear for the consequence clause [32] and (ii) the system shown in equation 2, which features free premise and consequent parameters (Figure 4A, right). Consequently, the fixed-linear setup rendered 4 free parameters, as opposed to 6 in the adaptive-constant and 8 in the adaptive-linear. The three systems were trained using the JNK-c-Jun measurements in control conditions –the reference model- and resampled versions of the same dataset (Figure 4B). Resampling was performed following the method known as bootstrapping introduced by Efron [33] creating 100 new datasets with 8 data pairs drawn with repetition from the JNK-c-Jun control observations. Model flexibility was calculated by simulating the resampled models and computing the standard deviation at each observed data point across models (Figure 4B). Fixed-linear and adaptive-linear setups varied greatly in their trajectories, adapting to resampled datasets and their differences with the dataset used to train the reference model (mean_σ_(fixed-linear) = 0.188 and mean_σ_(adaptive-linear) = 0.186), while adaptive-constant setup showed less flexibility to adapt to the resample data (mean_σ_(adaptive-linear) = 0.156). These results were reproduced when performing the analysis on the 3 additional datasets acquired upon treatment with MAPK inhibitors (see Table S1). The adaptive-constant setup showed a noteworthy loss of accuracy, while no such loss was observed when comparing the fixed-linear model to the adaptive-linear model, indicating that most of the information on the data is captured by the consequent parameters. The estimated parameters in the adaptive-linear model were close to the fixed ones in the fixed-linear (see data-derived sensitivity analysis in materials and methods).

The high flexibility of the chosen fixed-linear setup could indicate over-fitting, which is typically assessed via cross-validation by splitting the experimental data into a training and a test set: when a modeling method performs well on a training set but yields a large test set error, this indicates that the patterns found in the training do not exist in the test data, and are due to random chance rather than true properties of the function that one seeks to estimate [34]. We sought to assess over-fitting using leave-one-out cross-validation by splitting the eight JNK-c-Jun pairs into two sets, where a single pair observed at a given time point was used for test and the remaining time points were used for training. A gFIS was fit to the training set and a prediction was made for the excluded observation using its JNK value. This process was performed for all eight data pairs, yielding eight predictions for the same number of differently trained models. Next, a gFIS was trained to the full dataset, creating a reference model that was used to make a prediction for each observed JNK value. The predictions of the reference model largely correlated with the test predictions (R = 0.89, Figure 4C), confirming model predictivity for this subset of experimental data (see Table S2 for the parameters estimated for the training models and the reference model).

After cross-validation and seeing that neither flexibility nor accuracy seemed to be compromised, we concluded that the parameter reduction strategy rendered a valid system encoding the nonlinear behavior of a signaling intermediate or transcription factor as a function of the upstream activity. Next, this method was used to model the dynamic behavior of the full network containing the 10 above-mentioned measurements upon 4 experimental scenarios.

### Prior knowledge and literature-based definition of initial prior knowledge network to determine training subsets

**Figure 5:**
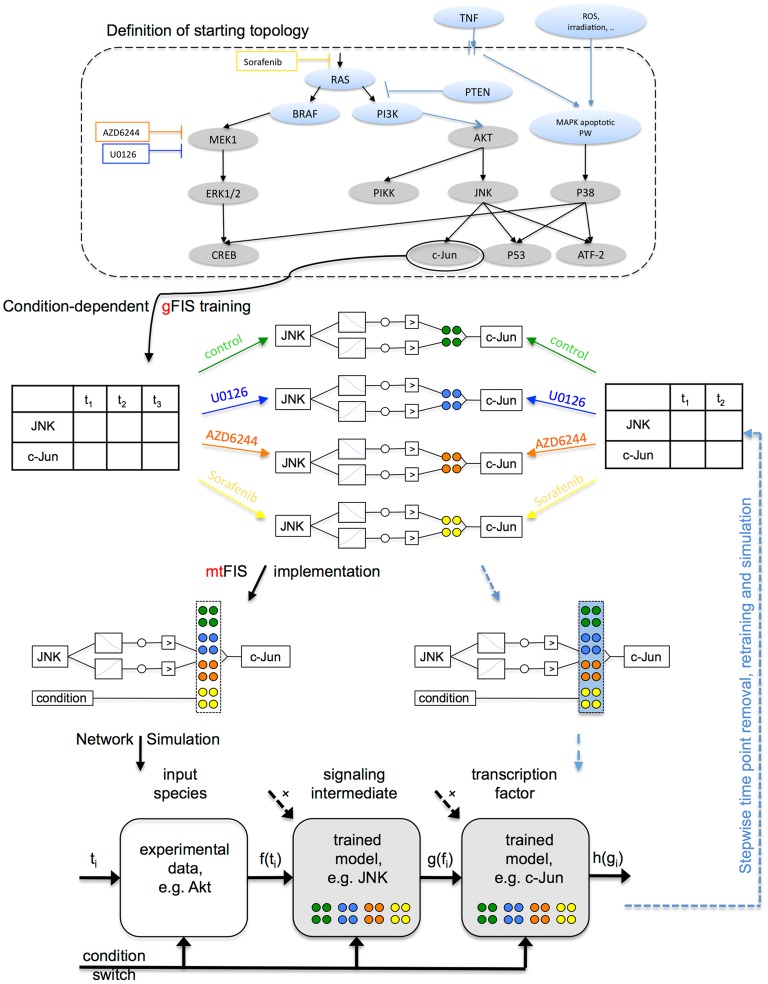
Workflow for network definition, fitting and simulation. Definition of starting topology. The initial step was the implementation of a network topology. Grey nodes are measured in our experimental assay. Blue nodes are not modeled or measured and are depicted here to enable understanding of network as a whole. Edges represent directed regulatory interactions reported in literature, and hence can represent activation or inhibition of the target. **Condition-dependent gFIS training.** A gFIS for each signal measured was trained to the corresponding dataset independently for each condition acquired. This enabled determination of the parameter set specific for each condition. **mtFIS implementation.** The condition-dependent parameters were used to create a multi-treatment model, including a naive condition switch to enable choice of parameters in the simulation for each condition. **Network simulation.** Upstream species could not be fitted to further upstream regulators. Hence, an input node consisting of a mapping function specified the measured value of the upstream species at the simulated time point. Thereby, the propagation of time as a signal was enabled. In turn, the fitted models were evaluated at the upstream-simulated value. The discontinuous black arrow represents the possibility of additional signaling intermediates upstream of each model. **Network evolution.** Blue dashed arrows indicate the workflow steps that can be repeated for a subset of the acquired data points up to a defined time point. See main text for the simulation resulting for models fitted to 96, 72 and 48 hours.

First, a network topology based on prior knowledge derived from literature was assembled (Figure 5, Definition of starting topology). Table S3 references the sources used for each interaction included. The network was used to determine the training data subset for one gFIS for each signal experimentally measured and subsequently for simulation. For training, the three perturbation and one control experiment parameters were fitted independently, yielding four sets of model parameters (Figure 5, Condition-dependent gFIS training). Thereafter, these condition-dependent parameters were used to create a multi-treatment model (mtFIS). A naive condition switch was included in the multi-treatment FIS equation to enable choice of corresponding parameters in the simulation of each specific treatment scenario (Figure 5, mtFIS implementation). See materials and methods for details.

Finally, once all multi-scenario models in the signaling network were defined, simulation of full network behavior was performed. For the readouts positioned highest upstream in the network such as Akt, a model could not be created due to lack of experimental measurements upstream that serve as input for model training. Therefore, to simulate the full network, a mapping function was used to determine the value of upstream readouts at each observed time point. Subsequently, propagation of the signal at the same time points was achieved by evaluating each downstream model with the simulated output of their upstream models (Figure 5, Network simulation).

### Analysis of model evolution

**Figure 6:**
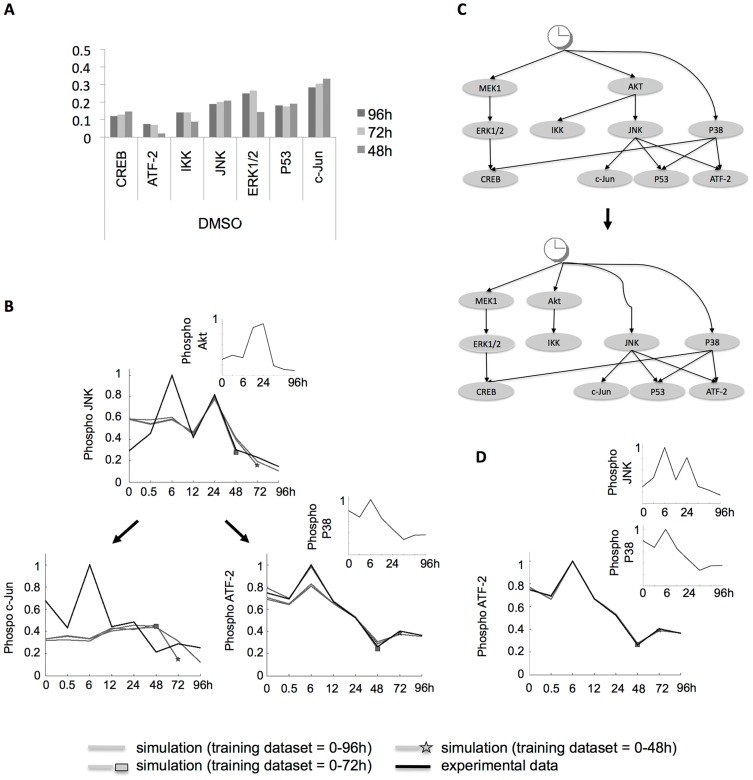
Model-suggested reimplementation of topology addresses emerging behavior. (**A**) Upon network simulation at the experimental time points, RMSE was calculated for models fitted to training measurements up to 48, 72 and 96 hours. No readouts were measured upstream of nodes Akt, P38 and MEK1, hence these were not trained. The model c-Jun shows a high error already at 48 h and no improvement over time in control conditions (see Figure S1 for all models). (**B**) Plotting c-Jun simulation (trajectory of input species in smaller time course) in the context of the signaling network for the models trained to measurements up to 48, 72 and 96 h (grey curves; a star and a square indicate the last training data point used in 72 and 48 hour models respectively) shows that the reason for failed simulation is the error propagated from JNK, which in turn could not be modeled as regulated by Akt, because the experimental data (black line) are indeed not related. On a neighbor branch of the prior knowledge network, ATF-2 successfully reproduces the behavior of P38 but is also suffering from error propagation from Akt to JNK. (**C**) To account for this emerging behavior, literature search suggested an alternative topology in which Akt signaling is parallel to JNK. (**D**) The reimplemented topology corrected JNK-regulated c-Jun simulation (see Figure S2 for all models) and as there was no error propagation from JNK, simulation of ATF-2 (grey lines) was then able to correctly follow P38 signaling.

We next sought to identify potential signaling rearrangements. Therefore, we created new training data subsets by removing late time points in a stepwise manner, i.e. new datasets contained only measurements from 0 to 72 h and from 0 to 48 h respectively. The training process was iterated (Figure 5, dashed blue arrow), including simulation of the newly trained network. Figure S1A shows the simulations of all models trained to the full and two reduced datasets. Figure S1B–E shows the RMSE resulting from evaluation against the training data. We observed that models with a RMSE below 0.2 accurately captured the trend in the data, and hence we defined the models above the 0.2 threshold as suggesting a disagreement between model simulation and experimental data. For those models fitted to experimental data from the control condition with a high error already at the period 0–48 hours, this indicated that the topology was likely wrong. This was e.g. the case for JNK (error 96 h = 0.18921 vs error 72 h = 0.20094) and c-Jun (error 96 h = 0.28357 vs. error 72 h = 0.30519; Figure 6A). Plotting the trajectories for those models and data as implemented in the context of the literature based network suggests that the reason for the large error for JNK and c-Jun is that in our experimental system Akt does not regulate the activity of JNK and c-Jun (Figure 6B), which is in contrast to the assumed model topology.

We then modified the starting topology to account for the emerging mismatch between model simulations and experimental data. Vivanco et al. postulated that the JNK signaling pathway is itself a functional target of PTEN in prostate cancer cells [35], suggesting that Akt and JNK can be activated independently from each other. Hence, we implemented JNK as being regulated independently from Akt by turning it into an input node (Figure 6C). The activity of c-Jun was then strongly associated to JNK (RMSE for c-Jun at 96 h = 0.12241, see Figure S2). Note that the larger RMSE of ATF-2 at early time points shown in Figure 6B could thus be reduced as well (Figure 6D).

It is noteworthy that, upon simulation of the full network, the error at each node features two components, i.e. (i) the topological error and (ii) the error propagating from simulation of the upstream layer. Therefore, in the specific case of the above-described modification of the network topology that led to improved simulation, the rectification in model misbehavior could be due to the transformation of a third level network component into a second level node, because a second level node receives as direct input the experimental data instead of the simulated value of the upstream model. Thereby, potential error propagation is prevented. However, we empirically show that the propagation error is not as great as that of the topology in e.g. the case of CREB upon U0126 (see Figure S2), which is downstream of 3 levels of regulators and still shows a remarkably accurate fit.

### Different mechanisms of action of two specific MEK inhibitors

**Figure 7:**
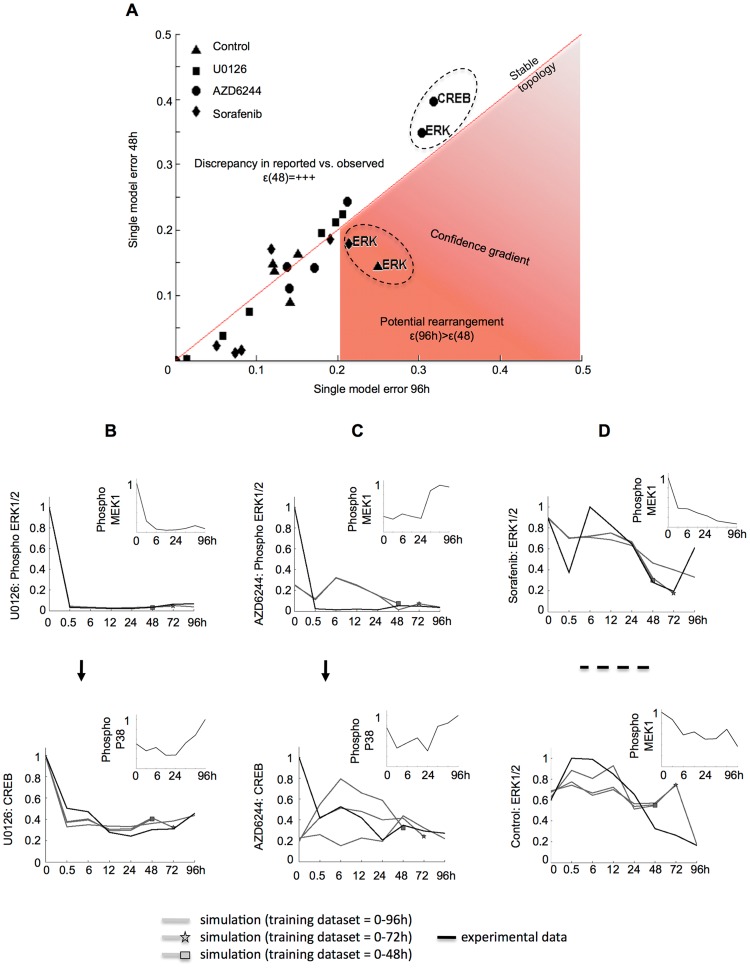
Analysis of error evolution with upgraded topology suggests A375-specific signaling rearrangement. Here, we implement the modeling concept illustrated in Figure 2F. (**A**) Error evolution displayed as errors for models trained with 0–96 h dataset (abscissas) against errors for models trained with 0–48 h dataset (ordinates). Each point represents a model trained to experimental data upon DMSO (triangle), U0126 (square), AZD6244 (circle) and Sorafenib (diamond). All models with errors at 96 h and 48 h below 0.2 RMSE capture the trend in the data (see Figure S2 for simulations and error calculations). Hence, models above 0.2 RMSE suggest a topological disagreement. The diagonal shows the models, whose errors exhibited no change upon retraining and simulation. CREB and ERK1/2 upon treatment with specific MEK1 inhibitor AZD6244 show a high error both at 48 h and 96 h, indicating that a disagreement in MEK1-ERK1/2 regulation is present from the first acquirement onwards. This disagreement is not present with specific MEK1 inhibitor U0126. The region of confidence constrains the models that have simultaneously a low error at 48 h and a high error at 96 h. This is the case of ERK1/2 both in control conditions and Sorafenib, which suggests a potential rearrangement in the canonical MAPK pathway. (**B**) Plotting ERK1/2 trajectory for retrained simulations and experimental data supports the suggestion by the model that MEK1 regulation follows canonical pathway implemented in the prior knowledge network upon treatment with specific inhibitor U0126, but (**C**) not upon specific MEK1 inhibitor AZD6244, indicating differential mechanisms of action of both inhibitors. (**D**) Trajectory plot for ERK1/2 confirms lack of regulation by MEK1 throughout early and late time points upon treatment with both Sorafenib inhibitor (upper panel) and control conditions (lower panel). The observation that canonical MEK1-ERK1/2 regulation is present upon U0126 suggests that he rearrangement is upstream of ERK1/2, and as supported by literature it could be specific in A375 melanoma cell line.

The study of network evolution using the newly implemented topology revealed poor model fits in ERK1/2 regulation upon treatment with MEK inhibitor AZD6244 (RMSE for ERK1/2 at 96 h = 0.30386) but not for U0126 (RMSE for ERK1/2 at 96 h = 0.013945; Figure 7A). This error then propagated to CREB. Taken together, this indicated that the non-canonical regulation of ERK1/2 by MEK1 suggested by the model was due to the presence of AZD6244, as revealed comparing the trajectories of ERK1/2 downstream of MEK1 in the literature-based network for U0126 (Figure 7B) and AZD6244 (Figure 7C). This is consistent with the fact that the phosphorylation level of a kinase is not always a proxy for its activity. Indeed, Wilhelm et al. showed that U0126 is a specific MEK1 inhibitor, which prevents its phosphorylation [36], while AZD6244 was developed by Yeh et al. [37] and reported as an inhibitor of ERK1/2 phosphorylation by selectively inhibiting enzymatic activity of MEK1. Its mechanism of action is documented in [38].

### Non-canonical MAPK signaling and Sorafenib-mediated topology in the A375 melanoma cell line

Following the modeling concept illustrated in Figure 2F, analysis of error evolution led to the identification of ERK1/2 (RMSE at 96 h = 0.2141 vs. RMSE at 48 h = 0.17851) as a potential rearrangement upon Sorafenib treatment (Figure 7A). The ERK1/2 trajectory upon Sorafenib treatment showed that the reason for this model prediction was the late activation of ERK1/2 at 96 h in spite of the decreasing activity of its regulator MEK1 (Figure 7D, upper panel). However, a disagreement was also shown to arise at 72 h in control conditions (Figure 7D, lower panel) and was also observed at 6 h in both control and Sorafenib (Figure 7A and 7B). The disagreement indicated by the model in control conditions suggested a non-canonical MAPK signaling network architecture, which could be further characterized. Sorafenib is an inhibitor of RAF kinases and VEGFR-2 and, thereby, prevents phosphorylation of MEK1/2 [36]. Because RAF is upstream of MEK1 and ERK1/2 [39], [40], the MEK1-independent increase of ERK1/2 activity at 6 h and 96 h upon Sorafenib treatment was unexpected. However, the A375 melanoma cell line used in this study has been shown to be more resistant to apoptosis than other melanoma cell lines. For instance, it has been shown that Sorafenib down-modulates the levels of Bcl-2 and Bcl-XL, and such down-modulation was shown to be MAPK-independent in A2058 and SKMEL5 melanoma cells but not in A375 cells [41]. Taken together, these observations suggest (i) a non-canonical signaling mechanism in the A-375 MAPK signaling network, and (ii) a Sorafenib-mediated effect. The signaling mechanism could be a new link upstream of MEK1, consistent with the observed ERK1/2 response upon MEK1 specific inhibition with U0126. Such mechanisms have been reviewed in Figure 1. For instance, scaffolding proteins have been shown to mediate crosstalk with other pathways [10]. In turn, the Sorafenib-mediated effect could be an off-target effect of Sorafenib on the crosstalk. Alternatively, the 96 h model disagreement could suggest that Sorafenib affects the crosstalk through a signaling rearrangement dynamically acquired to enable tumor proliferation at 72–96 h, in accordance with findings that up-regulation of resistance genes can arise within days of the start of the treatment.

### Benchmarking mtFIS implementation and simulation against a previous approach based on manually calibrated fuzzy logic modeling

To assess the performance of mtFIS, we compared the performance of our method with an already established fuzzy logic based modeling approach [16], where each model in the signaling network was manually implemented and calibrated, i.e. without parameter fitting. Our method was used to implement each node in the benchmark network and subsequently the data presented in [42] was used for automated parameter estimation, as detailed in Text S1. Figure S3A shows our simulation together with the benchmark simulation and experimental data. Figure S3B shows the RMSE of both simulations, revealing that high accuracy was achieved with our method. With the exception of IKK upon EGFR stimulation, all signals were more accurately encoded via mtFIS training and simulation. This is not a direct method comparison; instead, it specifically illustrates (i) a gain in performance when using our automated parameter reduction and training approach as compared to manually implemented fuzzy logic modeling, and (ii) the readiness of this work to be adapted to other datasets. In addition, benchmarking revealed limitations of our method in terms of data requirements, or in other words the ability to extend the number of inputs to a model, as described in Text S1.

## Discussion

In this work, we present a data-derived method to elucidate topological changes in experimentally measured signaling networks and pathway rearrangements that grant certain tumors their plasticity. We developed a training setup to fit the parameters of a Fuzzy Inference System (FIS) to experimental time-defined measurements. To increase interpretability, the number of qualities that are traditionally trained in a fuzzy inference system was importantly reduced. As part of this parameter reduction strategy, the contribution of the model features to the flexibility and accuracy of the output was calculated. Thereby, we could conclude that circumventing training of a number of model qualities without greatly compromising accuracy is possible. The number of free parameters was reduced to below the threshold of our 8 data points per condition acquired in our dataset per signaling intermediate or transcription factor measured. This pipeline rendered a generalized FIS that we termed gFIS. Based on literature, we defined a signaling network containing our experimental measurements, and subsequently a gFIS could be trained to the data corresponding to each model in the network. Next, a multi-treatment model (mtFIS) was created for each single experimental measurement that could reproduce its nonlinear behavior upon the 4 conditions of our experimental setup.

Evaluation of the network's performance revealed a mismatch in the MAPK stress response pathway, specifically in Akt-JNK-c-Jun as reported by Vivanco et al. and Aikin et al. [43], [44]. This mismatch was found in control conditions, indicating a context-dependent topology different from the canonical pathway implemented as initially found in literature. By introducing an alternative topology reported in the literature [35], we could take this emerging behavior into account, thereby improving the simulation. However, this manual literature search and implementation of modified network motifs can introduce a bias and be tedious when upscaled. We anticipate that methods that assume a prior knowledge network and then automatically optimize the topology of the network to identify signaling rearrangements that improve fit to data will be a key advance. In principle, this might be achieved via a combination of the objective function used here with the one presented by Saez-Rodriguez et al., which was developed to assemble Boolean logic models from a prior knowledge network and determine the optimal topology by quantifying the difference between data and global simulation while penalizing model size [15]. We acknowledge that there should be more rigorous definitions of the optimization process to account for models fitted to different number of time points to analyze network evolution than the ones used here. Hence, we propose that the exploration of the objective function mentioned above would be highly interesting.

Analyzing the evolution of the selected topology revealed a non-canonical architecture of the MAPK pathway in the A-375 melanoma cell line and a Sorafenib-mediated effect: while Sorafenib is a BRAF kinase and VEGFR-2 inhibitor and, thereby, prevents phosphorylation of MEK1/2 [36], the observed phosphorylation profile of ERK1/2 was not consistently inhibited. This lack of regulation of ERK1/2 by MEK1 was also present in the model in control conditions, suggesting crosstalk to the MAPK signaling in the cell line used in this study. This crosstalk regulator could be an off-target of Sorafenib or a signaling intermediate enhanced as a resistance mechanism, the latter being consistent with the late time point of the modeling disagreement. For instance, it has been reported that A375 melanoma cell lines show higher resistance to apoptosis than other melanoma cell lines, where anti-apoptotic proteins are down-modulated in a MAPK-independent manner, contrary to A375 [41]. Additionally, the analysis of model evolution described here led to observation of different mechanisms of action of the two specific MEK1 inhibitors used in this work. Such differential mechanisms were consistent with literature. Taken together, the fact that specific targeting of MEK1 led to consistent inhibition of ERK1/2 indicated that the above-mentioned A375-specific crosstalk should be upstream of MEK1.

To further evaluate our method, the dataset presented in [42] was used to benchmark it against the method described by Aldridge et al. [16]. Thereby, it was possible to show that our strategy for parameter reduction and automated model training increases performance over manual model implementation. In addition, due to the establishment of the general process for automation of model building, the model can readily be adapted to different datasets without demanding unavailable knowledge required to manually parameterize the MFs. On the limitations side, benchmarking revealed that the high accuracy and reusability of our method came at the cost of increased number of parameters and density of experimental data points for their estimation.

When modeling signal transduction, would it be possible to differentiate between transient versus sustained activation using our approach? To include the role of time in network topology, we based our method on the assumption that the behavior of each single node was the consequence of the behavior upstream of it. Therefore, to simulate the behavior of the whole network, only the upstream models were simulated on the first step, and its predicted value was used to propagate the signal throughout the signaling cascade for every simulation step. The role of time in logic models has been reviewed in depth elsewhere [45], [46]. It has been shown that transient ERK1/2 activation in PC12 cells upon EGFR stimulation by EGF induced proliferation, whereas sustained ERK1/2 activation by NGF induced differentiation [47]. In the future, data-derived logic models should be a key step forward to facilitate identifying the events in which duration of activation i.e. transient versus sustained is critical for cell signaling decisions. In principle this should be straightforward to achieve by including time as input analogously to the above-shown inclusion of signaling intermediates as inputs, which in turn would increase the constraints regarding dataset density. Thereby, direct quantification of the contribution of time as a signal would be enabled.

The exploration of network evolution described here suggested a Sorafenib-mediated effect that could be characterized, but the specific rewired interaction could not be identified and its cause could range from genetic mechanisms such as mutations to tight spatio-temporal pathway regulation (see Figure 1). To elucidate the mechanisms of topological complexity, more heterogeneous datasets combining phosphoproteomics with other data should provide a stronger basis. For instance, single-cell imaging data might facilitate understanding of spatio-temporal pathway regulation [48]. In previous work, we have used an exhaustive search of trained fuzzy models to identify nonlinear relationships in heterogeneous measurements of mitochondrial morphological, apoptotic, and energetic states by high-resolution imaging of human breast carcinoma MCF-7 cells [19]. This raises the possibility that further exploration of data-derived logic and other modeling approaches fitted to heterogeneous datasets should yield valuable insights into the sources of the mechanisms granting specific tumors its plasticity.

## Materials and Methods

### Multiplex measurements of phosphorylated and total protein concentrations

#### Cell culture

A375 melanoma cells were grown in RPMI 1640 medium supplemented with 10% fetal calf serum, sodium pyruvate, pen/strep and L-glutamine (Invitrogen, USA). MEK1/2-specific inhibitor U0126 was solved in DMSO (stock solution, 10 mM), and used at a final concentration of 10 µM. One pill of the multikinase inhibitor Sorafenib, targeting BRAF in melanoma cells was solved in 31,4 ml DMSO (stock solution, 10 mM) and used at a final concentration of 5 µM. MEK1/2-specific inhibitor AZD6244 was solved in DMSO (stock solution, 10 mM) and used at a final concentration of 3 µM. Measurements were acquired at 0 min, 30 min, 6 h, 12 h, 24 h, 36 h, 48 h, 72 h and 96 h. 0.1×10^6^ cells per sample were seeded in 6-well plates, one 6-well plate for each time point. Medium was discarded prior to addition of drug-containing medium for the given time points. DMSO was used as solvent control for the inhibitors.

#### Luminex-based analysis of total and phosphorylated proteins

We used the cell lysis kit from Bio-Rad (Hercules, USA) for the preparation of tumor cell lysates. After treatment, cells were washed by ice-sold PBS and lysed using 125 µl lysis solution according to the manufacturer's instructions Protein concentrations were determined using the Pierce BCA Protein Assay Kit (Thermo Scientific). To allow for the comparison of drug perturbations as well as comparison between cell lines, we detected the lowest protein concentration within this set of samples (100 µg/ml), and adjusted all other sample concentrations accordingly, using the phosphoplex assay buffer for dilutions. 50 µl of lysates containing 5 µg protein were then used for total and phosphoplex analyses according to the Bio-Rad protocol. At least 50 beads for each analyte region were collected for each lysate. Analysis was performed using Bio-Rad Manager6.0. Reported median fluorescence intensity (MFI) values for each analyte were used as a measure for the total or phosphorylated protein content in the samples. The following phosphorylation sites were detected using the Luminex machine: MEK1^Ser217/221^, ERK1/2^Thr202/Tyr204/Thr185/Tyr187^, Akt^Ser473^, GSK3b^Ser21/9^, c-Jun^Ser63^, JNK^Thr183/Tyr185^, p38MAPK^Thr108/Tyr182^, ATF-2^Thr71^, CREB^Ser133^, IKBa^Ser32/36^.

### Parameter reduction strategy and model implementation

In fuzzy logic, membership functions (MFs) allow transformation of the experimental data, thereby enabling the use of logic rules. To parameterize input and output MFs, two types of fuzzy logic systems have been widely used for inference, namely Takagi-Sugeno models [32] and Mamdani or linguistic models [49]. While the output MFs are constrained to constant or linear in a Takagi-Sugeno model, the output MFs in a Mamdani model are generalized to fuzzy sets. To enable the process of parameter estimation, the Takagi-Sugeno framework was used in this work.

#### Fixing number of rules and value of premise membership function parameters

The number of rules was fixed by considering only the combinations of low sets with themselves and analogously for high sets. Choosing rules with a combination of similar behavior, e.g. low and low, for multiple inputs was a simplification that yielded increased interpretability, since subsequently the degree to which each input contributes to the output could be extracted during the fitting process and is directly represented by the parameters of the consequent MFs. However, unlike kinetic rates in a physicochemical differential equation, some qualities of a FL model can be relatively abstract. Examples of abstract qualities are the number of rules, the width of a premise MF or, even more in a Takagi-Sugeno model than in a linguistic model, the consequent parameters. This renders the interpretability of the specific values of these qualities difficult in spite of the model reduction.

Because output parameters cannot be shared in Takagi-Sugeno fuzzy inference systems, we chose to fix the parameters of all input MFs to a generalized gauss function. In the case of low sets, the center of the function was set at a = 0 and in the case of high datasets at b = 1, which means that input measurements of 0 and 1 fully belong to the low and high input MFs, respectively (i.e. the degree of membership to the set low 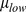 of a 0 input in Figure 3A equals 1 and 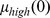 equals 0, and conversely to a 1 input 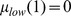 and 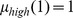). For both cases, we took 0.4247 for the width 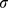, as this is the default MATLAB standard. The effect of fixing said parameters was assessed by resampling the training dataset in fixed versus free setups (see data-derived sensitivity analysis).

#### Model implementation

MATLAB Release R2011b, The MathWorks Inc., Natick, Massachusetts, United States, was used for model implementation, fitting and simulation. Equation 2 and 3, i.e. a gFIS for single and another for double regulated intermediates, were implemented as a MATLAB function. Another function was implemented to fit them using an unconstrained nonlinear optimization process. Due to the flexibility of the fuzzy inference system, the solver converged to solutions which where very accurate at the data points but out of range between them. In turn, poor interpolation power led to large error propagation when the signal of upstream models was propagated to simulate downstream models. To correct for this by selecting those fits with the best interpolating power, 20 equidistant points were synthesized to evaluate the model between the data points and a Euclidean penalty was calculated by taking all distances for those simulations over 1 and below 0, which we respectively termed *PositiveOffset* and *NegativeOffset*. This penalty was then used to punish the root-mean-square error calculated in the objective function 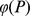 as shown in equation 4 for a given vector 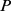 containing the parameters calculated by the optimization algorithm:
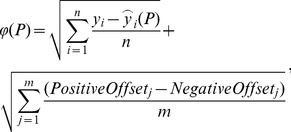
(4)where *n* is the number of data points, *m* is the number of interpolated i.e. synthetic points, 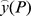 is the simulation calculated by the model at a given data point and 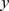 is the experimental measurement at the same data point. The use of the number of data points for the root mean squared calculation of the model error served as simple means to account for number of training data points when comparing models during network evolution.

To prevent the fitting process to be trapped in local optima, all values for initial parameters were randomized following a uniform distribution.

Finally, a MATLAB structure formatted as a Takagi-Sugeno fuzzy inference system (e.g. P53.fis) was created with rules and input MFs parameterized as mentioned above and the different parameters resulting of the fitting process as output MF parameters for each rule. All other FIS qualities were implemented as default in MATLABS's Fuzzy Logic Toolbox.

### Data-derived sensitivity analysis

The reference model for the fixed-linear setup (Figure 4A, left) was implemented as previously shown. The adaptive-constant setup (Figure 4A, center) was implemented by creating a structure consisting of a zero-order Takagi-Sugeno FIS, i.e. the input MFs were Gaussian and parameterized as mentioned above and the output MFs were set as *constant*. Fitting was performed via a combination of the least-squares method and the backpropagation gradient descent method for training FIS in the Fuzzy Logic Toolbox. Analogously, the adaptive-linear setup (Figure 4A, right) was implemented by creating a structure consisting of first-order Takagi-Sugeno system and using the same fitting process. The bootstrapped predictions were calculated by submitting the three above-mentioned “create and fit system” functions to bootstrapping for 100× resampling with repetition, a method introduced in [33]. All models are shown in Figure 4B, and standard deviation was calculated for the prediction at each data point. As a signature of model flexibility in terms of impact of training dataset in the simulation at each data point, the mean of the standard deviation across models is displayed.

As a signature of accuracy, the error 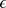 was calculated as root-mean-squared error for all models and deviation 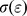 was calculated across errors. This analysis is displayed in Figure 4B for control conditions and extended to all conditions in Table S1.

Comparing the estimated premise parameters in the adaptive-linear model to the fixed ones in the fixed-linear model revealed high similarity (a = 0, b = 1, 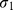 = 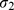 = 0.4247 in the fixed-linear model, while a = 0.0223, b = 1.019, 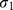 = 0.431 and 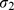 = 0.427 were estimated for the adaptive-linear setup with an error of 0.122). However, other values yielded estimated premise parameters similar to those new values, indicating a bias of the training algorithm towards the initial parameters and confirming that the consequent parameters outweigh the premise in their contribution to accuracy. This bias had no effect in our approach because the premise parameters were fixed.

### Implementation of multiple perturbation models

The free parameters for a gFIS corresponding to each species in the signaling network were fitted to the experimental data separately for each condition. To compile all parameters in a multi-treatment trained FIS at no increased parameter cost, two Boolean functions were introduced to represent absence 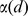 and presence 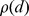 of each drug 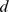, which we together termed naive condition switch as shown in equation 5 and 6:
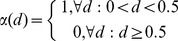
(5)

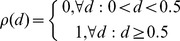
(6)Automation of the model building process was achieved by modifying the above-mentioned MATLAB function to “create system and fit it” to include the naive drug switches. Loosely speaking the 4 naive condition switches are simple Boolean functions added to all rules of each FIS, so that upon simulation they are evaluated at a value with the sole purpose of outputting either 1 or 0, thereby neglecting the parameters learned in the conditions that are not currently simulated. Equation 5 and 6 were included in the code by adding 1 input with 2 trapezoidal MFs parameterized as Boolean functions for each one of the 3 pharmacological inhibitors.

Formally, as shown in Figure 3, the parameter reduction strategy yielded 2 rules per system per dataset. Hence, to include all parameters trained for the 4 datasets, the multi-treatment model for each species in the signaling network consisted of 8 rules. Consider for illustration the model 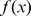 encoding the transcription factor c-Jun, which according to starting topology in Figure 5 is regulated by one single intermediate, namely JNK. Following equation 2, the model including the 3 naive condition switches 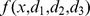 for a value 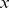 of JNK is shown in equation 7:
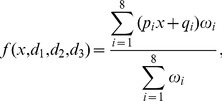
(7)where 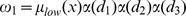, 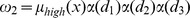, 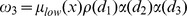, 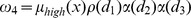, 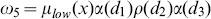, 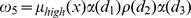, 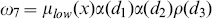, 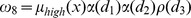, 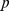 and 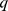 are the fitted parameters and 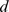 is a discrete value that represents each drug and will let the switches output zero when absent (equation 5) and one when present (equation 6) for each drug in order to neglect all but the parameters learned in the conditions being simulated. This approach is valid for our experimental setup, where treatment consists of single drugs. It can easily be adapted to other experimental scenarios, such as multiple drugs present simultaneously, and can be implemented using MATLAB's graphical interface for the Fuzzy Logic Toolbox. For more details on the simulation process, the reader is referred to the next section.

### Full network simulation

For model simulation, values were defined for each naive condition switch corresponding to the current simulation. Simulation was performed by automatically assigning to each upstream node the experimental value measured at a given time point. No model was implemented for the upstream nodes because no experimental measurement was acquired upstream of them that could serve as input. An exception to this was the benchmark simulation, where the upstream nodes are models implemented to encode their behavior as a function of time, analogously to the benchmark method. Subsequently, models downstream were evaluated, thereby propagating the signal via the SIMULINK model.

The network states in a logic model can be updated in a synchronous and deterministic manner or asynchronously [45]. Here, the state of each node in the network was synchronously determined by the state of the nodes upstream at a specific time point.

## Supporting Information

Figure S1 **Time-defined model simulations against training subsets and corresponding errors according to initial prior knowledge network.** (**A**) Simulation of full network. Each species in the network is shown as rows for each treatment used (columns). Simulations are shown in grey upon training to data subsets containing measurements from 0 to 96 h, up to 72 h (indicated with a star) and 48 h (indicated with a square). Experimental measurements are shown as black lines. (**B–E**) Root-mean-squared error was calculated for each simulation for all treatments. Control conditions show a high error for the Akt-JNK-c-Jun pathway implemented in the prior knowledge network. This observation led to literature-based reimplementation of the network topology (see Figure 6).
(TIF)

Figure S2 **Time-defined model simulations against training subsets and corresponding errors according to reimplemented signaling network to account for emerging behavior.** (**A**) Simulation after reimplementation of the initial prior knowledge network. Each species in the network is shown as rows for each treatment used (columns). Simulations are shown in grey upon training to data subsets containing measurements from 0 to 96 h, up to 72 h (indicated with a star) and 48 h (indicated with a square). Experimental measurements are shown as black lines. (**B–E**) Root-mean-squared error was calculated for each time-defined simulation for all treatments, enabling analysis of network evolution. This analysis revealed a potential rearrangement upstream of ERK1/2, which could be specific for A375 melanoma cell line. Additionally, high error in ERK1/2 simulation upon AZD6244 but no U0126 suggested differential mechanism of action of the two specific MEK1 inhibitors (see Figure 7).
(TIF)

Figure S3 **Benchmarking our method shows high reproducibility, reduced prior knowledge demanded for model parameterization and increased reusability at the cost of large increase of data requirements.** (**A**) Model simulation (grey line) shows up to 10 fold increase of accuracy with respects to benchmarking method (blue line) and an improvement in capturing the trend over the 24 hours of measurement in the benchmarking data (black line). This improvement was enabled by the described modeling approach over the 4 conditions out of 10 in the benchmark dataset (columns) selected to enable comparison. The application of the approach presented here to an additional dataset revealed that parameters can be readily estimated, thereby, easing the process of model implementation and simulation to encode the behavior of a signaling network. This is an advantage over methods that require manual model implementation. (**B**) RMSE calculated for our method (grey bars) and the benchmark method (blue bars) corroborate the increased accuracy of the method presented here. RMSE was normalized to the max for each simulation. Challenges revealed by benchmarking in terms of data density requirements are extensively described in Text S1.
(TIF)

Table S1
**Extension of data-derived sensitivity analysis to all experimental conditions.** The analysis resampling the data to evaluate flexibility and accuracy of the approach, as described in Figure 4 for control conditions, was performed for all conditions. The model implementation and training setup featuring fixed premise parameters and adaptive linear consequent parameters was contrasted to two established setups: (i) A system with adaptive premise parameters and adaptive zero-order consequent parameters and (ii) a system with adaptive premise parameters and adaptive linear consequent parameters, i.e. the same setup as the fixed-linear system with the difference that here the premise parameters were not fixed. For each condition, a reference system was created which was trained to all data points acquired in said condition. The RMSE 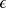 was calculated for the reference model as a metric of accuracy. The training data set was resampled 100 times via bootstrapping and the standard deviation of the simulations at each data point was calculated as a metric of flexibility (first raw for each modeling approach). The error of every bootstrapped model was calculated, and the deviation for all 100 models is shown (second raw for each setup). Ranking the calculations for the metrics of the setups for each condition shows that the first in accuracy (third raw for each modeling approach), i.e. the setup with least references error is the adaptive-linear closely followed by the fixed-linear. The most robust setup is the adaptive-constant, and the fixed-linear and adaptive-linear perform similarly, indicating that neither accuracy nor flexibility are greatly compromised by using the parameter-reduced setup. See main text for further details on the resampling rationale.(XLS)

Table S2 **Parameters estimated for the reference and cross-validation JNK-c-Jun models.** In order to discard over-fitting we performed a leave-one-out cross-validation approach. The JNK-c-Jun control dataset featuring eight data pairs was split into a test set, consisting of one excluded observation, and a training set consisting of the remaining observations. A gFIS was fit to the training set and a prediction was made for the excluded observation using its JNK value. This process was performed for all eight data pairs, yielding eight training set models. Next, a gFIS was fit to the full dataset, creating a reference model. The parameters shown in equation 2 for the reference model are shown in the first row. Subsequent rows are the train set models for each excluded time point. See main text for details on cross-validation. The final row shows the parameters estimated during bootstrap using the adaptive-linear setup.
(XLS)

Table S3 **References used to determine interactions used for model fitting.** Left column indicates the relationship between a regulator and its downstream target. Interactions can be direct or indirect, the latter being trough intermediates not measured, hence not included. Right column indicates the source where the corresponding relationship was found. During model implementation process, reported interactions indicated which data subset to use as input e.g. regulatory kinases and which data to use as output e.g. regulated transcription factor for each model fitted in the topology. An overview of the pathway map can be found online at the KEGG database http://www.genome.jp/dbget-bin/www_bget?map04010.
(XLS)

Text S1 **Benchmarking methodology and limitations.**
(DOC)
