# Supplementary figures and images for: Data-Derived Modeling Characterizes Plasticity of MAPK Signaling in Melanoma
Source: PLoS Comput Biol. 2014 Sep 4;10(9):e1003795. doi: 10.1371/journal.pcbi.1003795 (PMC4154640; doi:10.1371/journal.pcbi.1003795)

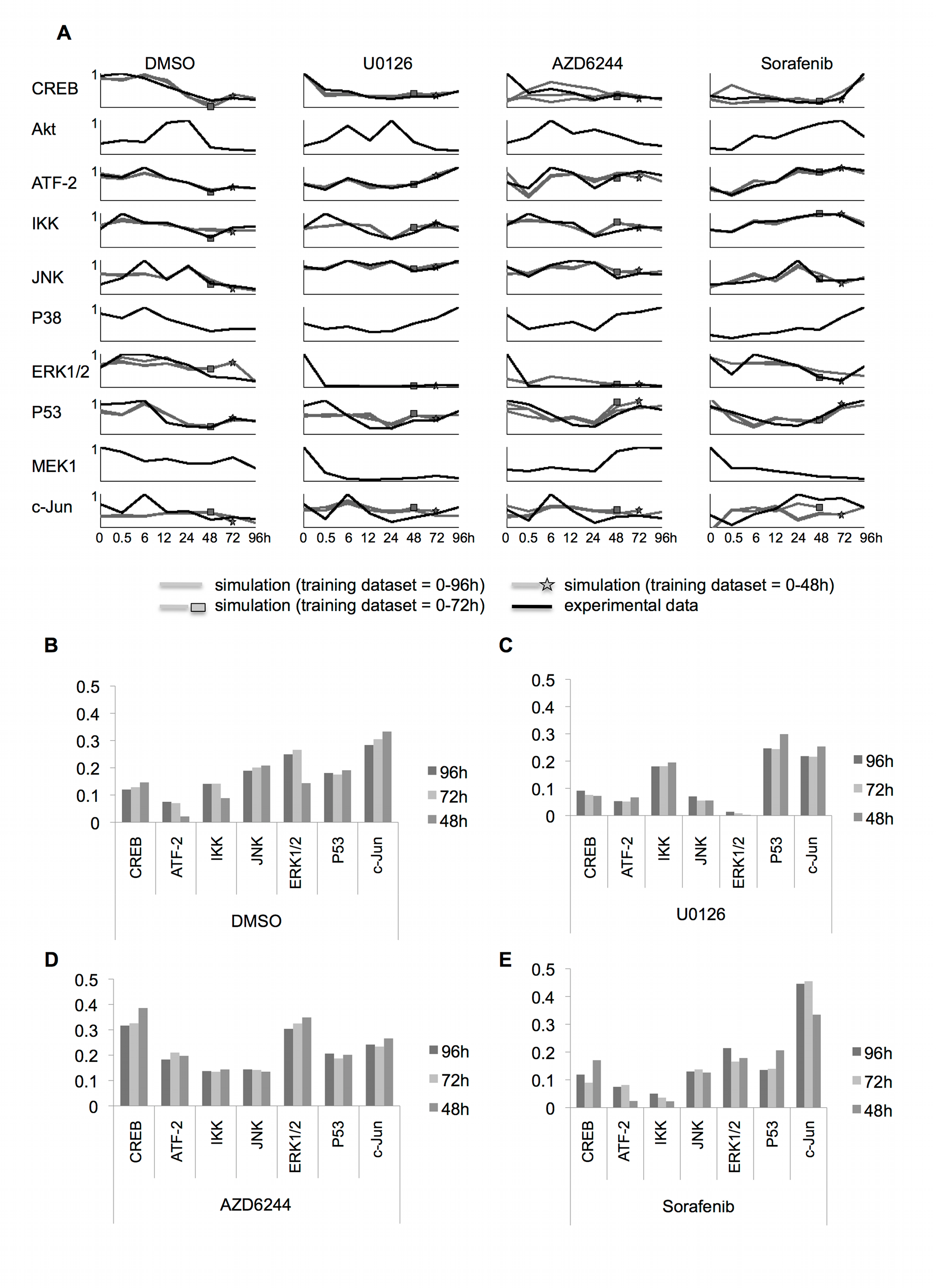

Supplement: Figure S1 — Time-defined model simulations against training subsets and corresponding errors according to initial prior knowledge network. (A) Simulation of full network. Each species in the network is shown as rows for each treatment used (columns). Simulations are shown in grey upon training to data subsets containing measurements from 0 to 96 h, up to 72 h (indicated with a star) and 48 h (indicated with a square). Experimental measurements are shown as black lines. (B–E) Root-mean-squared error was calculated for each simulation for all treatments. Control conditions show a high error for the Akt-JNK-c-Jun pathway implemented in the prior knowledge network. This observation led to literature-based reimplementation of the network topology (see Figure 6). (TIF) [file pcbi.1003795.s001.tif]

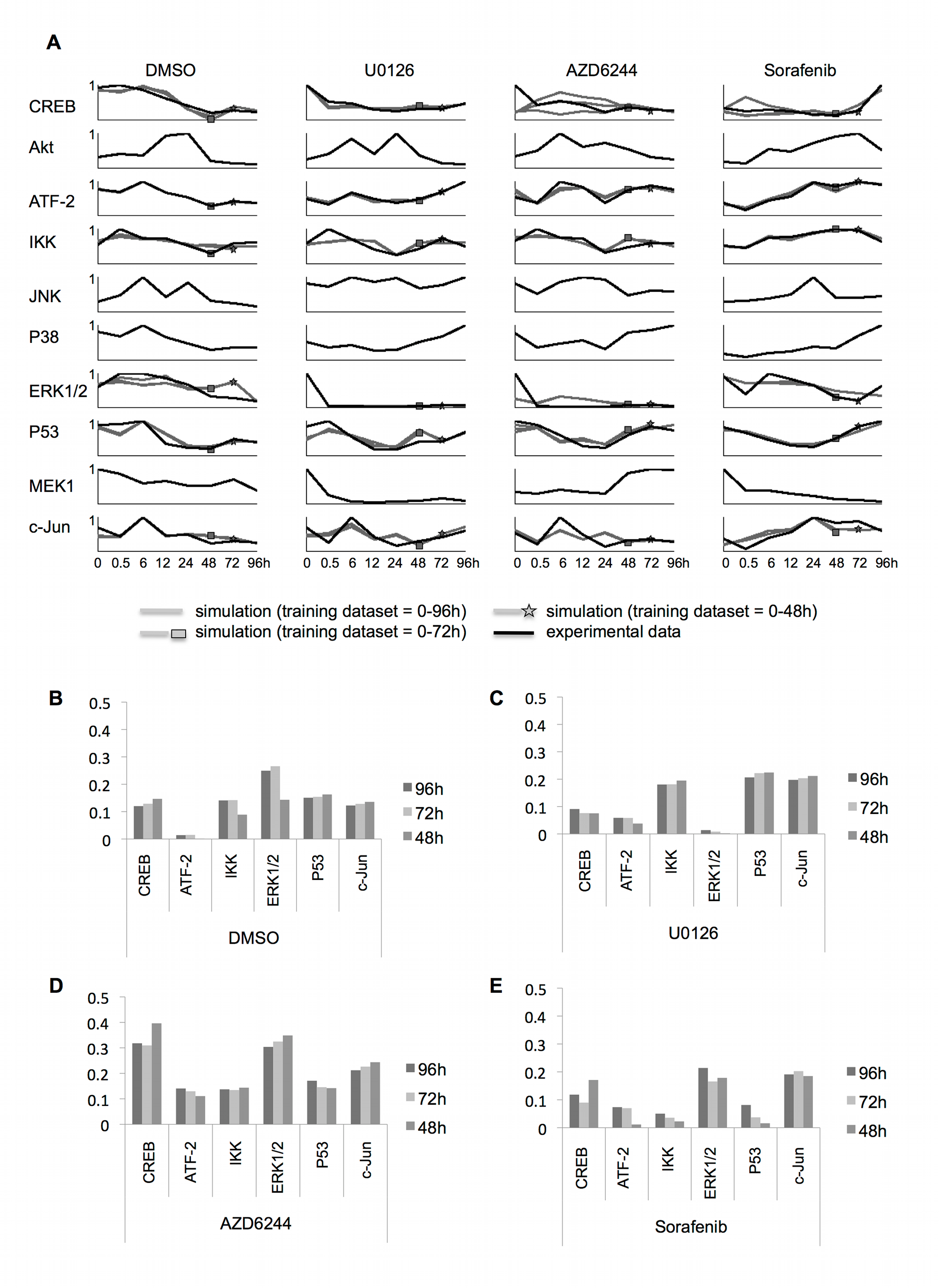

Supplement: Figure S2 — Time-defined model simulations against training subsets and corresponding errors according to reimplemented signaling network to account for emerging behavior. (A) Simulation after reimplementation of the initial prior knowledge network. Each species in the network is shown as rows for each treatment used (columns). Simulations are shown in grey upon training to data subsets containing measurements from 0 to 96 h, up to 72 h (indicated with a star) and 48 h (indicated with a square). Experimental measurements are shown as black lines. (B–E) Root-mean-squared error was calculated for each time-defined simulation for all treatments, enabling analysis of network evolution. This analysis revealed a potential rearrangement upstream of ERK1/2, which could be specific for A375 melanoma cell line. Additionally, high error in ERK1/2 simulation upon AZD6244 but no U0126 suggested differential mechanism of action of the two specific MEK1 inhibitors (see Figure 7). (TIF) [file pcbi.1003795.s002.tif]

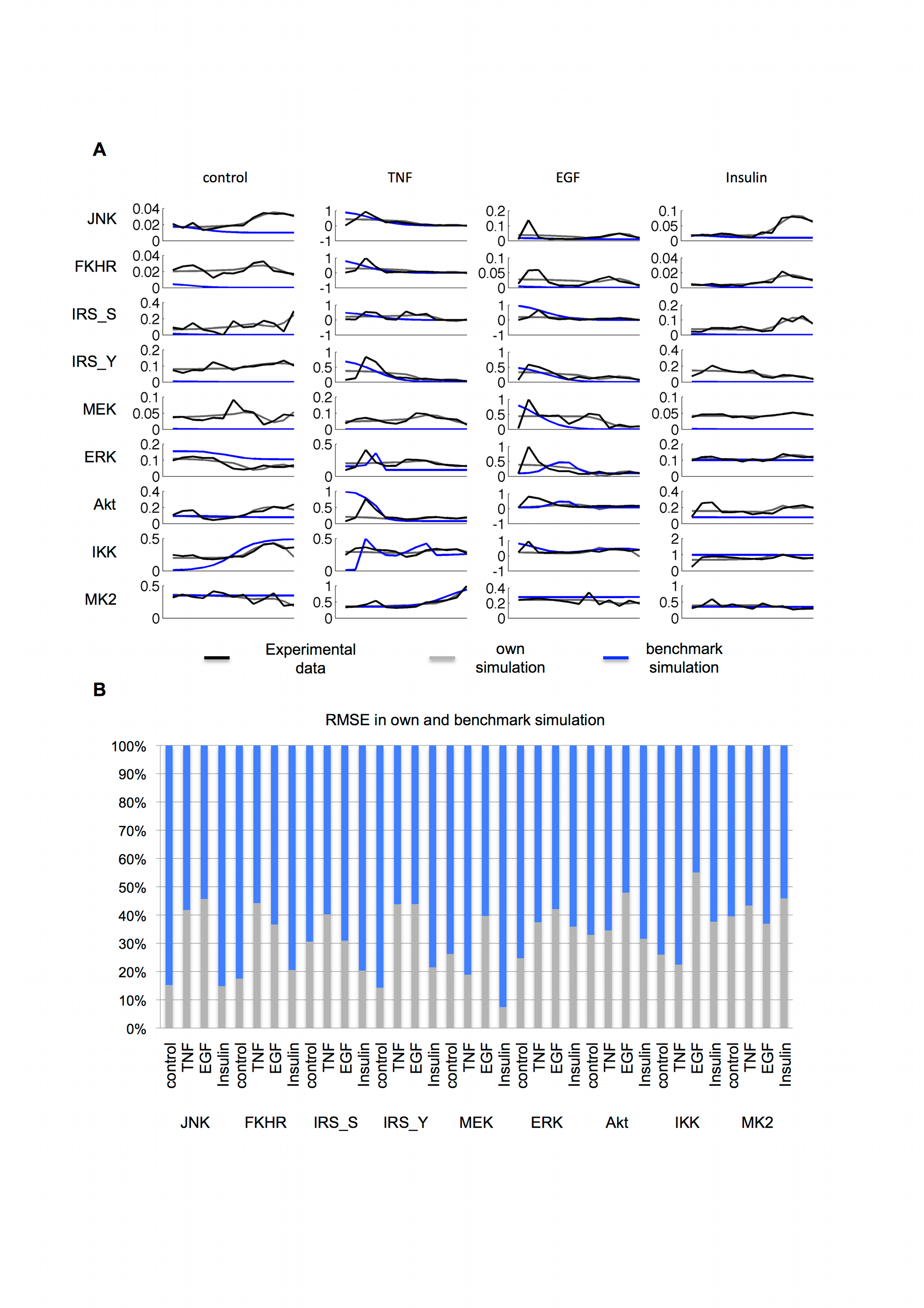

Supplement: Figure S3 — Benchmarking our method shows high reproducibility, reduced prior knowledge demanded for model parameterization and increased reusability at the cost of large increase of data requirements. (A) Model simulation (grey line) shows up to 10 fold increase of accuracy with respects to benchmarking method (blue line) and an improvement in capturing the trend over the 24 hours of measurement in the benchmarking data (black line). This improvement was enabled by the described modeling approach over the 4 conditions out of 10 in the benchmark dataset (columns) selected to enable comparison. The application of the approach presented here to an additional dataset revealed that parameters can be readily estimated, thereby, easing the process of model implementation and simulation to encode the behavior of a signaling network. This is an advantage over methods that require manual model implementation. (B) RMSE calculated for our method (grey bars) and the benchmark method (blue bars) corroborate the increased accuracy of the method presented here. RMSE was normalized to the max for each simulation. Challenges revealed by benchmarking in terms of data density requirements are extensively described in Text S1. (TIF) [file pcbi.1003795.s003.tif]
