# Supplementary material for: Data-Derived Modeling Characterizes Plasticity of MAPK Signaling in Melanoma
Source: PLoS Comput Biol. 2014 Sep 4;10(9):e1003795. doi: 10.1371/journal.pcbi.1003795 (PMC4154640; doi:10.1371/journal.pcbi.1003795)
Supplement: Text S1 — Benchmarking methodology and limitations. (DOC) [file pcbi.1003795.s007.doc]

**Text S1**

**Benchmarking methodology**

In our experimental setup, inhibitors were used at single concentrations and were therefore either present or absent. Conversely, the dataset presented in [42] was acquired in 10 combinations of different concentrations of TNF/EGF/Insulin. To enable comparison in same conditions, 4 datasets were selected: measurements acquired in control conditions and maximum conditions of each single treatment, i.e. (i) 100 ng/mL TNF, no EGF and no Insulin, (ii) no TNF, 100 ng/mL EGF and no Insulin and (iii) no TNF, no EGF and 500 ng/mL Insulin. For the selected datasets, we calculated the RMSE corresponding to the simulations presented in [16] (see Figure S3A and S3B). In the benchmark method, a SIMULINK model was implemented to represent and simulate the logic gates (see supplementary data in [16]). In our method, the inputs implemented to account for different concentrations of treatment in the SIMULINK model were removed, since only maximum concentration datasets were selected. Additionally, some changes needed to be implemented in the topology of the SIMULINK model. IRS(Y), IRS(S), Akt, ERK, MK2, JNK, FKHR and IKK were implemented analogously to the benchmark model. The modeling of feedback loops is not addressed in the method presented here, hence the feedback ERK-MEK was removed. Consequently, no delay and max gates were required, and hence casp8 and proc3 had to be removed. See materials and methods for more details on model simulation.

Following this topology, an mtFIS for each node in the network was trained to the corresponding data by using the model implementation and training process described above and in materials and methods. Subsequently, the SIMULINK model was simulated and RMSE was calculated. Figure S3A shows the experimental data and the simulation for each node in the network performed with both methods.

For a detailed description on how models were implemented to account for multiple treatments using naive condition switches, please see materials and methods, “Implementation of multiple perturbation models”.

**Benchmarking limitations**

The impact on model performance of our parameter reduction and automated model training was assessed by comparing mtFIS to the fuzzy logic based modeling approach presented in [16]. The results of the benchmarking are shown in Figure S3B. The method by Aldridge et al. could successfully encode experimental data measured upon 10 combinations of TNF/EGF/Insulin at different concentrations by including these treatments directly as inputs. Compared to our method expressing each intermediate solely as a function of its regulators, the direct inclusion of treatments increases the number of free parameters, which grow with the membership functions necessary to *fuzzify* these inputs and adds model qualities that need to be parameterized, namely the number of rules to accommodate these inputs and how these inputs are combined in the rules. Our method was designed to avoid requiring the prior knowledge necessary to parameterize these qualities. Could we include all treatments as inputs to all FIS models and train them, thereby learning how changing concentrations of treatments affect the behavior of their targets? We observed that this immediately increases the density of the dataset required: each individual species implemented in the model contains $2(n+1)$ free parameters, where $n$ is the number of upstream regulatory intermediates for this specific species defined in the prior knowledge network. For instance, to include a species regulated by 3 upstream signaling intermediates in a network measured upon 3 treatments, more than 2(6+1)=14 data points should be acquired in order to maintain the number of free parameters below the number of data points. This is a clear advantage of the benchmark method, in terms of the number of data points required and hence the ability to include additional inputs, when the knowledge or a hypothesis regarding the targets of these inputs is at hand.
